# Correlations between the Aging Behavior and Finite Element Method Simulation of Three Silicone Elastomers

**DOI:** 10.3390/ma17163961

**Published:** 2024-08-09

**Authors:** Svenja Marl, Xiaofei Ni, Tobias Hornig, Christian Spieker, Ralf-Urs Giesen, Hans-Peter Heim, Michael Fister

**Affiliations:** 1 Institute for Materials Technology, University of Kassel, 34125 Kassel, Germany; 2 Institute for Drive and Vehicle Technology, University of Kassel, 34121 Kassel, Germany

**Keywords:** silicone rubber, aging, FEM, Mooney–Rivlin model

## Abstract

The material parameters required to describe material behavior can change with the age of the components due to chemical and physical aging processes. The finite element method (FEM) is a tool for designing components for later use. The aim of this study is to correlate the change in the mechanical properties of silicone elastomers from standard tests over a longer period of time with the parameters of the Mooney–Rivlin model. To date, there are no publications on the development of the Mooney–Rivlin parameters of silicone elastomers over a storage period. For this purpose, the Shore A hardness, rebound elasticity, compression set and tensile properties were investigated over an aging period of approx. 200 days on two liquid silicone rubbers (LSRs) and one room-temperature-vulcanizing (RTV) silicone rubber. Depending on the silicone elastomer used, different trends in the characteristic values can be observed over the storage period. In general, increases in the Shore A hardness, rebound resilience and stress at a 100% strain with a decrease in the compression set can be determined. In addition to standard tensile tests, the material’s multiaxial behavior under tension was probed, and it was found that the similarly stress at a 100% strain increased. Finite element simulations verified the standard tensile test and corresponding Mooney–Rivlin model parameters. These parameters from the uniaxial tensile were validated in the multiaxial behavior, and the model’s accuracy in representing material properties and the influence of aging on the FEM simulation were affirmed.

## 1. Introduction

### 1.1. Silicone Rubber

A wide variety of products are made from silicone elastomers, e.g., sealings, damping and protective elements, as well as hoses. Depending on the area of application, the requirements for their processing and properties are different. For this reason, silicone rubbers are available in different groups. A distinction is made between room-temperature cross-linking silicone (RTV) and high-temperature cross-linking silicone (HCR). The HCR types can be cured in a range between 130 and 200 °C, and they are, in turn, subdivided into solid silicone rubber (HCR: high-consistency rubber) and liquid silicone rubber (LSR: liquid silicon rubber). HCR is mainly processed into continuous components (e.g., profiles or hoses) by extrusion. Possible curing reactions with HCR are peroxide cross-linking and addition cross-linking. LSR, on the other hand, is processed into molded parts (e.g., sealing rings) in the liquid injection molding process due to its lower viscosity, and it is cured exclusively via an addition reaction.

The room-temperature-curing silicones already cure at temperatures starting from 20 °C, and they are divided into one- and two-component systems. The one-component (1K) types cure via condensation cross-linking, in which low-molecular components are released. Water (e.g., humidity) is needed to start the curing. The two-component (2K) types can also cure via condensation cross-linking, but they can also cure via addition cross-linking. Compared to LSR, RTV has an even lower viscosity and can usually be processed in potting. One possible application for them is in the electronics industry as a potting compound for coils and transistors. Here, silicone is used as an insulator. Another advantage of addition cross-linking is that the cross-linking reaction can be accelerated by temperature (e.g., 80 °C).

### 1.2. FEM Simulation of Silicone Elastomers

Numerical simulations based on FE methods can be represented in three dimensions, and they show how components behave in relation to mechanical loads and temperatures [1]. For FEM simulations, material coefficients must first be determined on the basis of measurements, which are stored in the simulation software for elasticity, density, thermal expansion coefficients, thermal conductivities, etc. [2].

In most calculations for linear elastic materials, the Neo-Hookean law describes the stress–strain behavior. For silicone elastomers, this model cannot be used for all loading cases due to the strongly non-linear material behavior [3]. Therefore, the literature refers to the hyperelastic material models of, e.g., Yeoh, Ogden and Mooney–Rivlin [3]. Some of these material laws have been successfully transferred to silicone elastomers, as [4] shows. For material strains up to 100%, the Mooney–Rivlin model reproduces the stress–strain behavior of silicone elastomers well [4]. However, this material model, like the other models mentioned, does not take into account material aging. For the damage of rubber-like materials, which can also be described by hyperelastic material models, a damage approach can be used [5], whereby the mechanical damage progress is described by the exponential function that was detailed in [6]. Here, however, the damage due to aging is not taken into account and does not refer to silicones, which is why an explicit function is necessary for silicones for expansion. Another physical calculation model is described in [7]. This model gives good results for the description of the stress–strain behavior of filled rubbers. This model is a very interesting approach for the FEM since the velocity and temperature dependence are also described. Likewise, the modeling of filler-induced stress softening, as well as hysteresis effects, has already been carried out in [8]. It is necessary to investigate whether the transferability of these models can be applied to real-time aged silicones.

Material aging results from acceleration forces in the form of vibration and thermal stress. Depending on the place of use, very large temperature gradients occur in a motor vehicle [9]. At external temperatures of −30 °C during the winter, temperatures of over 200 °C can occur, e.g., at the turbocharger intake side or at the cylinder head gaskets.

In order to be able to carry out a meaningful FEM simulation, it is essential to select the right material models and to store the corresponding material data.

### 1.3. Aging of Silicone Elastomers

There is little in the literature about the aging of silicone elastomers. In addition, a distinction must be made between the different silicone types or cross-linking mechanisms. Essentially, silicone elastomers are very resistant to ozone and UV radiation, as well as weathering of any kind (even at higher temperatures). It has been stated that, even after years of use, no real damage forms, and the mechanical properties change only slowly [10]. For example, resistance to weathering was investigated in a long-term test in various regions of Australia (Melbourne, Innisfail, Cloncurry, etc.) over a period between 2 and 20 years. Ten different silicone elastomers, two condensation-curing RTVs and eight peroxide-curing HCRs were tested for their mechanical properties [11].

The silicone samples that have probably been stored the longest, according to the literature, were examined after 40 years. In the process, 14 mm high blocks of ten different elastomers were stored at three different locations (Shawbury, UK; Innisfail; and Cloncurry in Australia), and they were placed to avoid direct exposure to light and rain. The change in hardness over the thickness of the samples was also investigated. What is striking about the tests is that the RTV grades showed significantly worse values for tensile strength and elongation at break than the LSR grades used. Compared to the many HCR grades tested, the RTV grades showed slightly lower values for tensile strength and hardness. These statements can be made for all aging periods of 2, 5 and 20 years. RTV, HCR and LSR were also compared with each other in [12]. This involved investigating the Shore A hardness after storage for 55 days at different temperatures (20, 90, 135 and 175 °C). It was found that all materials show an increase in hardness of 10 to 15 Shore A from 20 °C to 175 °C, i.e., they demonstrate similar behavior. Unfortunately, the comparison to the unstored samples as well as an exact specification of the silicone type are missing [13]. Further studies on aging by radiation have been published on peroxide-curing HCR and condensation-curing RTV [14,15,16]. Among others, the influence of the filler (silica) of HCR on aging was investigated by electron radiation [16], and the influence of a combination of UVA radiation, temperature (60 °C) and 85% relative humidity over 16 days on the Shore hardness and compression set of HCR was investigated [14]. The condensation-curing RTV was aged by proton radiation, and the changes in the mechanical properties (tensile test and Shore hardness) and chemical degradation (GCMS and FTIR) due to radiation were determined [15].

The only investigations carried out in the field of aging by radiation in conjunction with simulations (FEM) were changes in the micro-contact area of silicone seals. It was found that the contact area of the seal to the substrate is reduced, although the pressure on the surface increases, because the silicone becomes harder and the compression set increases. Only the hardness and the compression set were determined as mechanical parameters. A contact model developed by Abaqus was used as the FE model [14].

Furthermore, there are studies by Keshavaraj et al. on the aging of various RTV silicones [17,18,19,20]. The silicones differ in their elasticity moduli and in their cross-linking mechanisms. The aging effects were investigated in connection with ozone and humidity, whereby different pH values were also set to simulate acid rain or cleaning agents. From the mechanical properties (tensile test), models of the changes in cross-link density were then calculated [17,18,19,20].

With thermal aging, the rubber elasticity is maintained for several thousand h even at high temperatures (200 °C). However, this depends on the chemical composition of the polysiloxanes. Polydimethylsiloxane is most resistant to purely thermal stress, while polydibenzylsiloxane, for example, is already degraded at this temperature [10]. A relatively recent publication from 2017 [21] deals with the mechanism of the thermo-oxidative aging of silicone rubber. It was found that over the aging time and temperature, the low molecular weight components (SiO_3_C and SiO_4_) increase, which indicates an oxidation of the side group and a cross-linking reaction. Furthermore, the compression set and the hardness of the silicone increase during aging. In addition, an FEM contact model was used to determine the influence of aging on the contact surface of silicone seals. The investigations are preliminary work to [14], whereby no other mechanical characteristic values were determined here apart from the hardness and the compression set [21].

A more serious problem in aging can be the influence of water vapor, which depolymerizes the polydimethylsiloxanes. This can be reduced by functional groups (e.g., phenyl or vinyl groups) [10]. Due to the high gas permeability of silicone, it is resistant to water as a liquid but unstable when exposed to water vapor.

During the storage of silicone rubber in various media, it was proven that aromatic and chlorinated hydrocarbons lead to swelling. Silicones are very resistant to aliphatic hydrocarbons and water [10]. For example, relaxation tests on LSR in air or water were already carried out by Cui et al., whereby it was shown that water only has a minor influence after a longer period of time [22]. Further studies on the storage of LSR in distilled water were carried out by Cui et al. and Ghanbari-Siahkali et al. Here, on the one hand, investigations were carried out on stress relaxation in connection with the time–temperature superposition principle, whereby an influence of the medium was demonstrated [23]. In addition, the degradation process in water was investigated over two years using DSC, TGA and FTIR [24].

Other researchers have also shown that silicones with different hardness react differently to deionized water and acids. Softer silicone swells more but loses less weight during a storage period of 30 days than harder silicone. However, the silicone type was not declared in more detail in [25]. Li et al. investigated the influence of acids on LSR, whereby no mechanical properties but only chemical properties such as material degradation were analyzed using FTIR and microscopy [26]. Acid rain also leads to strong material degradation in insulators made of silicone elastomers, as A. R. Verma demonstrated using energy-dispersive X-ray spectroscopy (EDX), thermogravimetric analysis (TGA) and FTIR [27]. The results obtained by Weltschev et al. show that the changes in mechanical properties during the storage of silicone rubber in fuel oil with 10% biodiesel at 20 °C are very slow and can be significantly accelerated by storage at a certain temperature (e.g., 40 °C) [28].

Zakaria et al. also conducted storage tests without thermal aging or the influence of media. Here, an RTV and an LSR were aged under tensile load for up to 3 months, whereby a slight reduction in elasticity was observed [29].

As a conclusion, it can be stated that the aging of silicones has already been investigated, but not in connection with the determination of characteristic values for simulation or apart from the source [28].

### 1.4. Literature Review

The amount of studies in the literature dealing with the problem of aging of silicone rubbers has been very limited in recent decades. There are general publications on real-time storage [11] from the 1990s, which also deal with all available silicone, including RTV, HCR and LSR. More recently, there have been some publications in the field of electrical engineering in which silicone elastomers were used for insulators. Here, however, only individual silicone classes, such as RTV, are examined. Often, only electrical properties are investigated with regard to aging. Mechanical properties are neglected [12,30,31,32]. There are further investigations in the field of fuel cells [26,33]. Here, silicone elastomers are examined for their atomic and mechanical properties in strongly acidic media. Studies on long-term aging at high temperatures with media are also few and far between. In [34], aging was carried out over 6 weeks at 195 °C in a functional oil. There are no publications on the real service life (more than 2 years) of silicone elastomers in applications such as automotive or medical technology, especially considering that the brand names of the silicones are well known.

## 2. Materials and Methods

### 2.1. Materials

Two LSR were used, namely Silopren LSR 2040 from Momentive Performace Inc. (Leverkusen, Germany) and KEG-2000-40 from ShinEtsu (Tokyo, Japan). Furthermore, an RTV, Neukasil RTV 230 with cross-linker A 149 from the company Altropol (Stockelsdorf, Germany), was used. Both liquid silicone and room-temperature curing silicone were investigated to cover a wide range of applications for addition-curing silicones with this study. As there is only a very limited database on the aging of addition-curing silicone rubbers, these were selected for the study. The LSRs used have a hardness of 40 Shore A, while the RTV’s specifications range between 28 (24 h) and 30 (7 d) Shore A depending on the curing condition [35,36,37]. To create better contrast when using visual measurement systems, the three materials were each colored with 1 wt% Elastosil Color Paste FL Black RAL 9005 F from Wacker Chemie AG (Burghausen, Germany) [38].

### 2.2. Manufacturing of Test Specimens

Four specimen geometries were fabricated for five different tests to analyze the material behavior over time. Cylindrical disk type A is used for the hardness test and the determination of rebound resilience. The two hot-cross-linking LSR materials were produced on an HM 65/130 screw injection molding machine from Wittmann Battenfeld (Kottingbrunn, Austria) that was converted for silicone processing. The single-cavity injection mold used has a needle valve nozzle, allowing for a precise injection volume to be realized even at low shot weights. Fluid temperature control cools the material to a constant temperature of 20 °C from the screw to the injection point of the cavity. The temperature of the injection mold was controlled using electrical mold heating circuits. The LSR was metered by means of a SilicoStar Mini from 2 Komponenten Maschinenbau GmbH (Marienheide, Germany) mixing and metering system. The base components A and B were pneumatically conveyed from two separate 1 L disposable cartridges to the mixing block, where the material was homogenized with the addition of color and then fed to the screw. The differences in the sample geometries ensured individual adjustment of the respective heating time and shot weight. Half of the LSR samples produced underwent post-curing for 4 h at 200 °C, since post-curing achieves complete cross-linking.

Table 1 below shows the injection parameters used for the respective sample geometries.

**Figure 1:**
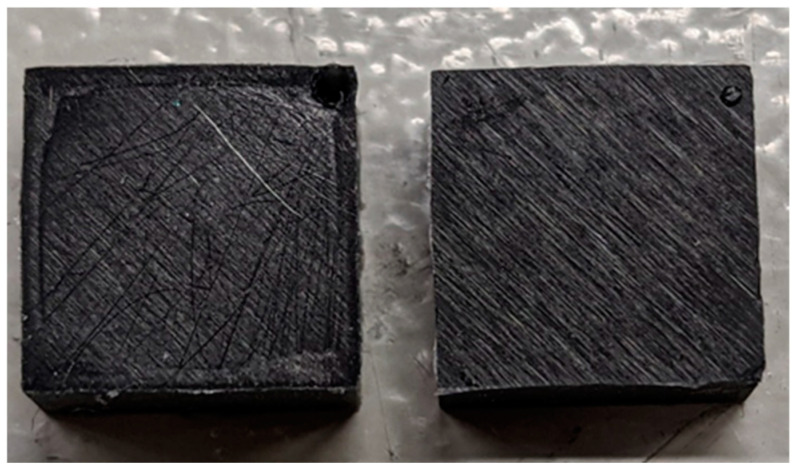
H sample. (**Left**): T_W_ = 180 °C; (**right**): T_W_ = 130 °C.

The geometry of the H sample corresponds to a square cross-section with an area of *l* × *b* = 10 mm^2^ and a depth of *t* = 5 mm [4]. Due to the particularly high accuracy requirements of the H specimen geometry, the vulcanization temperature was adjusted from 180 °C to 130 °C, as otherwise, specimen cracking occurred along the mold parting plane when the mold was opened (cf. Figure 1).

Plates of different heights were cast to produce the RTV specimens. The two-component material was weighed with the paint and mixed in a three-stage program (cf. Table 2) using a DAC 1000.2 VAC-P speed mixer from Hauschild (Hamm, Germany) and, in the last step, de-aerated under a vacuum to guarantee a bubble-free material. Subsequently, the RTV was directly cast to prevent early cross-linking.

After a vulcanization time of 24 h, the plates could be de-molded, and the required specimens could be punched out. In the case of the H specimen, punching out proved insufficient due to the dimensional accuracy requirements of this specimen geometry. Therefore, to obtain more accurate results, this geometry was cut from the RTV plate using a waterjet cutter. No annealing treatment was applied to the RTV specimens.

### 2.3. Test Procedure

To determine the influence of aging on the three silicones as comprehensively as possible, a total of five test procedures were used. In each test cycle, the five batches were subjected to the test procedures mentioned below.

#### 2.3.1. Tensile Test According to DIN 53504

To determine the change in the mechanical properties of the material, ten S2 shoulder bars per cycle and material were subjected to a tensile test in accordance with DIN 53504 [39]. For this purpose, the universal tensile test rig “Insepkt table 5 kN” from the company Hegewald und Peschke (Nossen, Germany) with a 500 N load cell was used, whereby a measuring accuracy of 0.001 N was achieved. The specimens were tightened to a preload of 0.1 MPa at a feed rate of 50 mm/min, and then the extensometer was applied, and the specimen was pulled at 200 mm/min until failure. The extensometer had an initial gauge length of 20 mm.

#### 2.3.2. Compression Set According to DIN ISO 815-1

According to DIN ISO 815-1 [40], the compression set is a parameter that describes the permanent deformation of an elastic material after the end of a defined compressive load. For this purpose, three specimens each were compressed by 25% and stored in the oven at 175 °C for 22 h. The specimens were then removed from the mold. After demolding, the specimens were left to relax for 0.5 h on a wooden board, after which the recovery was determined. The compression set can be calculated using Formula (1).

(1) $$DVR=\frac{h_{0}-h_{1}}{h_{0}-h_{s}}\times 100$$

*h*_0_ is the initial thickness of the specimen in mm.

*h*_1_ is the thickness of the specimen after recovery in mm.

*h_s_* is the height of the spacer in mm.

#### 2.3.3. Shore A Hardness According to DIN EN ISO 868

To record the changes in material hardness during the storage period, the Shore A hardness was determined according to DIN EN ISO 868 [41]. The “digitest II” hardness tester from Bareiss (Oberdischingen, Germany) was used for this purpose. The indenter, a hardened steel pin with a diameter of 1.25 mm, was pressed into the specimen, and the depth of the indentation was measured. Within each test cycle, five specimens were used for analysis.

#### 2.3.4. Rebound Resilience According to DIN 53512

The rebound resilience tester from Bareiss was used to investigate the rebound resilience according to DIN 53512 [42]. This test method allows the damping behavior of a material to be determined. For this purpose, a pendulum hammer strikes the surface of the test specimen from a defined height with a defined force. The rebound resilience results from the ratio of the rebound height to the initial drop height of the pendulum. Five specimens were analyzed in each case to ensure a meaningful sample. Type A cylindrical washers from the previous Shore A hardness measurements were used.

#### 2.3.5. The Storage and Aging of the Test Specimen

After production, the specimens were stored in a standard climate at 23 °C and 50% relative humidity. The test specimens were stored for a total of 200 days. Within the first month, the samples were taken and tested every seven days. The samples did not change visually during storage. After that, the interval between individual measurements was increased to two weeks for the RTV and the Momentive LSR 2040. Due to the high testing effort, the interval for the ShinEtsu KEG-2000-40 was increased to two months after the first month. Likewise, the interval for the H sample was increased to two months for all three materials after the first month due to the high effort required for sample preparation and testing.

#### 2.3.6. H-Tensile Test

**Figure 2:**
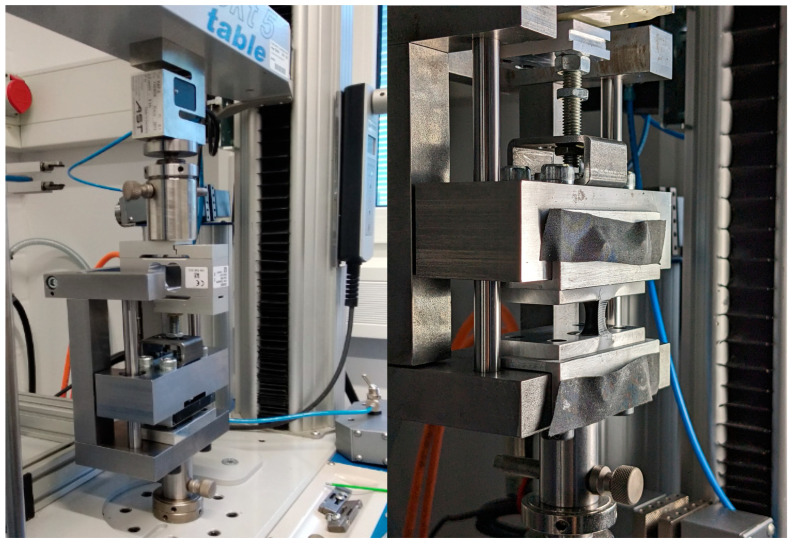
The test rig of the H-Tensile test (**left**); bonded LSR specimens (**right**).

Compared to the uniaxial tensile test, an H-Tensile test is designed with a square geometry [4]. The idea is a multiaxial stress test, whereby the square cross-section only allows for observation in one plane, with the assumption that both square surfaces behave in the same way. Figure 2 shows the test rig of H-Tensile testing and the H sample. The test was performed at “Inspekt table 5 kN” with a 500 N load cell and a constant test speed of *v* = 3 mm/min. An H specimen was located between the tension beam and the thrust bearing of the test fixture. The H silicone specimen with an area of *l* × *b* = 100 mm^2^ and a depth of *t* = 5 mm was tethered to two aluminum plates that were bolted to the thrust bearing and the tension beam. The movement of the transverse of the testing machine led to the advancement of the tension beam. During the advancement, the silicone specimen was exposed to a tensile force. The displacement of the silicone specimen was recorded by a digital image correlation.

Considering the preparation and storage of specimens during the aging test and their installation on the experimental equipment, the experimental equipment was improved from [4]. Some necessary pre-experiments were carried out to achieve the best possible pasting results. The prepared silicone specimen was bonded using adhesive to two aluminum plates that were ground and cleaned with isopropanol. It should be noted at this point that the bonding quality, to a certain extent, also influences the experimental results, so care should be taken to ensure a relatively high bonding quality here as well. For example, the adhesive should be applied to both the metal surface and the silicone surface so that the adhesives can be stable during the experiment. The application area of the adhesive on the metal surface must be larger than the cross-section of the specimen to seal the specimen. By using spacers and appropriate pins, the specimen can be well positioned (Figure 2). The specimens should be bonded 3 to 7 days before testing to ensure that the adhesive bonds well with the specimens and aluminum plates. The samples were applied with the help of white spray paint. A suitable spray paint should be selected so that applying a thin layer on the surface of the silicon specimen does not have an influence on the mechanical properties. Using a stencil grid with a mesh size of 0.3 × 0.3 mm, the dot pattern can achieve optimum quality for digital image correlation.

## 3. Results

### 3.1. Tensile Test

#### 3.1.1. Tensile Strength

**Figure 3:**
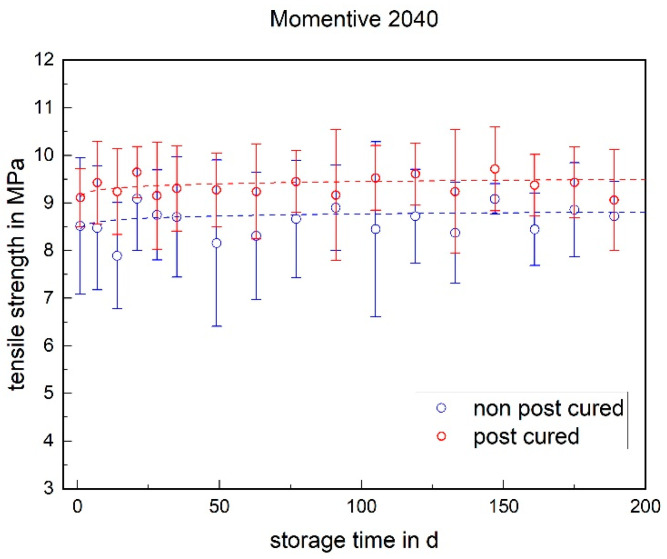
The tensile strength of Momentive LSR 2040 as a function of the storage time.

**Figure 4:**
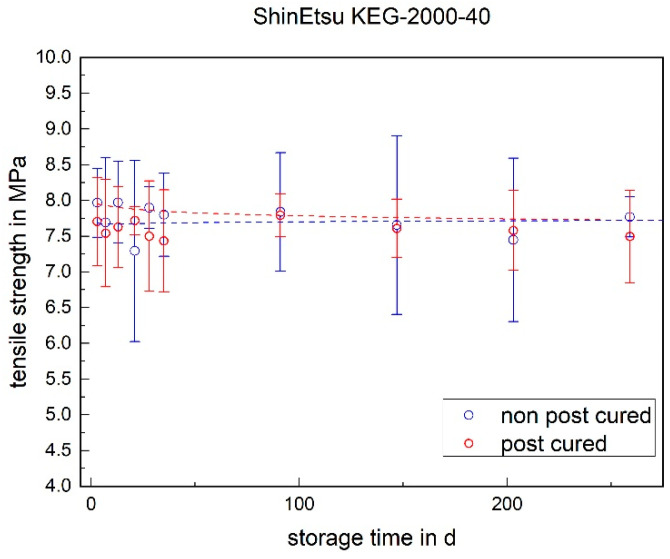
The tensile strength of ShinEtsu KEG-2000-40 as a function of the storage time.

**Figure 5:**
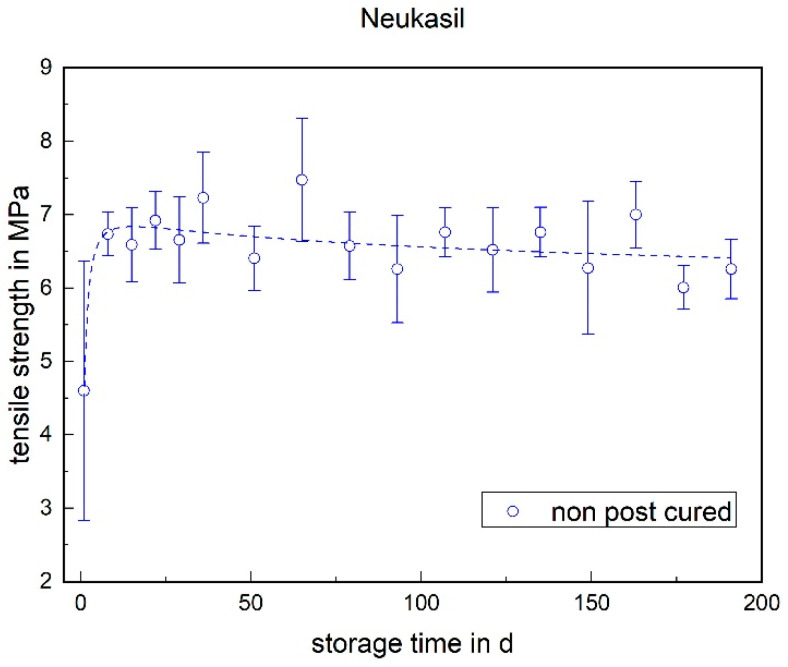
The tensile strength of Neukasil RTV as a function of the storage time.

The tensile strength of both LSRs remained almost constant over the storage period. No significant change could be detected (cf. Figure 3 and Figure 4). With RTV, however, the tensile strength increased significantly within the first week. Twenty four hours after potting, i.e., at the time of demolding, the tensile strength was 4.6 MPa and exhibited a very high standard deviation (cf. Figure 5). After one week, a tensile strength of 6.7 MPa was achieved. The tensile strength of the RTV tended to decrease slowly over the storage period. The increase within the first week can be explained by post-cross-linking. After 24 h, there was still no complete cross-linking.

#### 3.1.2. Elongation at Break

**Figure 6:**
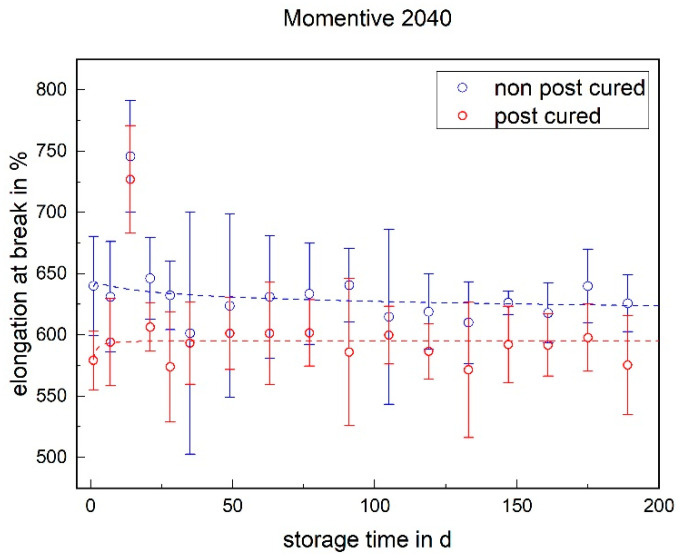
The elongation at break of Momentive LSR 2040 as a function of the storage time.

**Figure 7:**
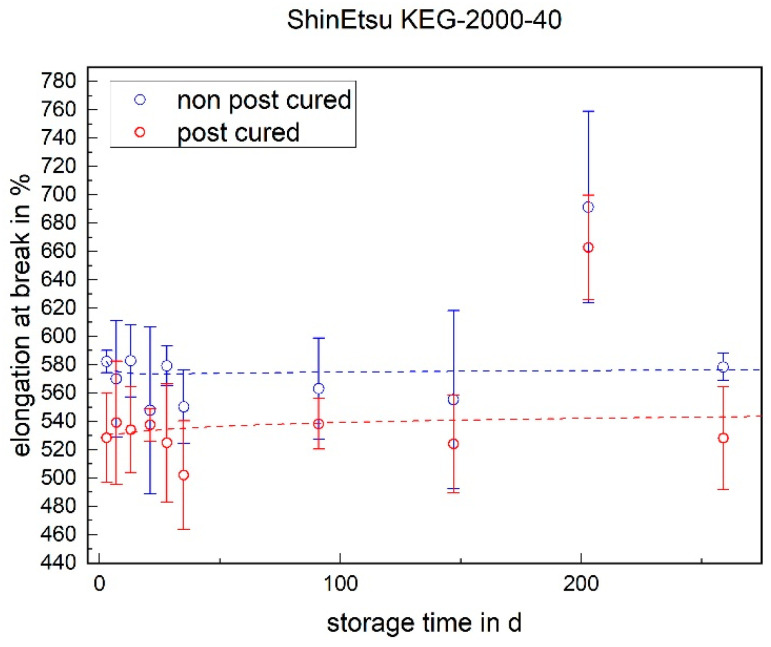
The elongation at break of ShinEtsu KEG-2000-40 as a function of the storage time.

For both LSRs, the non-post-cured samples exhibited a higher elongation at break than the post-cured samples. This effect was maintained over the entire storage time and can be explained by post-curing during the post-cure process. No significant change in elongation at break was observed for LSR over the storage period (cf. Figure 6 and Figure 7).

**Figure 8:**
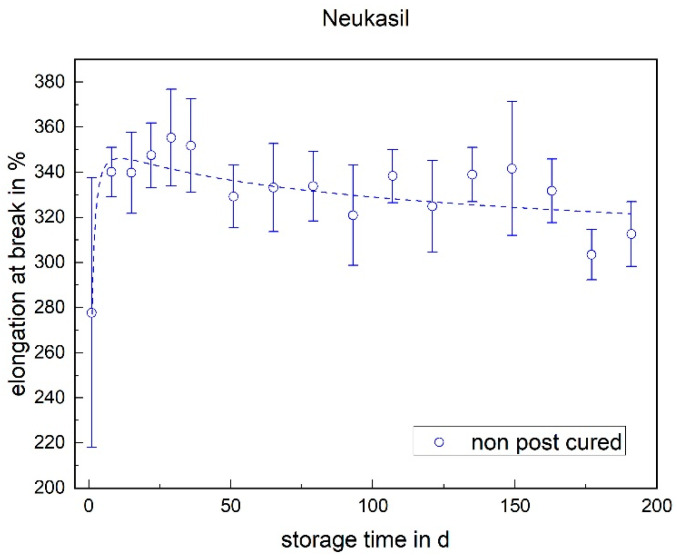
The elongation at break of Neukasil RTV as a function of the storage time.

The situation was different for RTV (cf. Figure 8). As already observed for the tensile strength, the elongation at break after demolding was significantly lower than the elongation at break after 7 days. An increase in the elongation at break of approx. 60% (absolute) can be measured within the first week. After the first week, a slight decrease can be observed in the storage period. This effect also occurred with the tensile strength, although it is more pronounced here.

#### 3.1.3. Stress at 100% Strain

**Figure 9:**
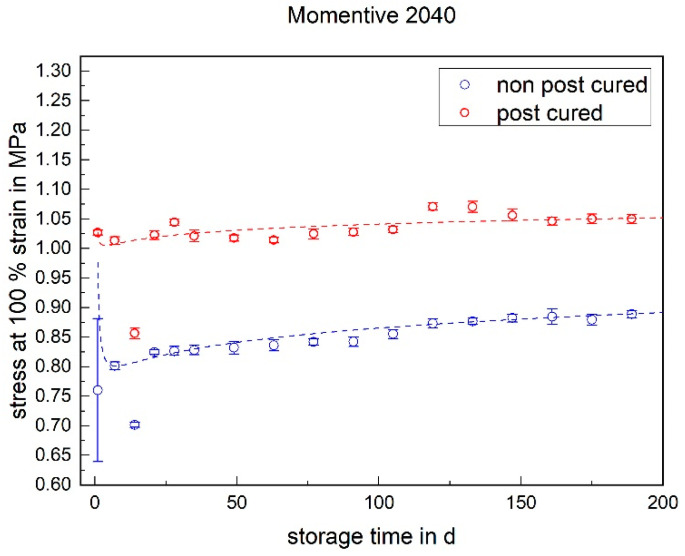
Stress at 100% strain of Momentive LSR 2040 as a function of the storage time.

**Figure 10:**
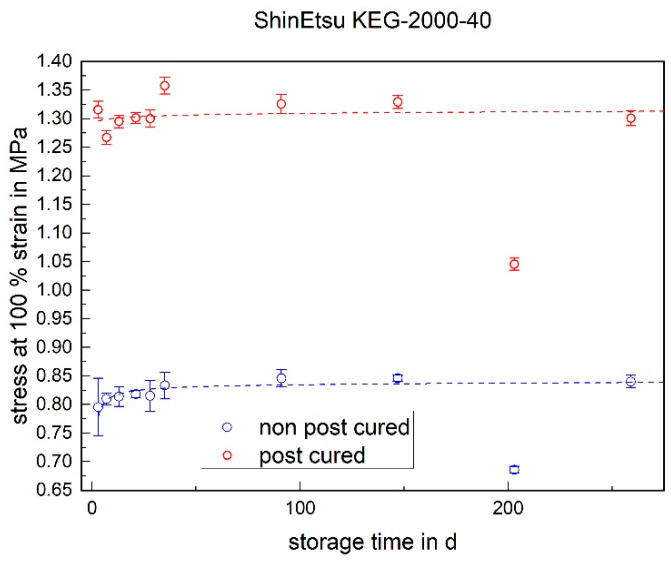
Stress at 100% strain of ShinEtsu KEG-2000-40 as a function of the storage time.

The stress at 100% strain is a value representing the elasticity of the material. For both LSRs, the post-cured samples have a higher stress value at 100% elongation than the non-post-cured samples (cf. Figure 9 and Figure 10). This means that the post-cured samples are less elastic than the non-post-cured samples. This effect can be explained by the post-cross-linking during the tempering process. More cross-linking points require a higher force for the same deformation (elongation), which increases the stress. For both LSRs, an increase in stress at 100% strain can be determined for the non-post-cured samples within the first few days. This increase is more pronounced with Momentive’s LSR 2040 and can be observed up to approx. 120 days. With the LSR KEG-2000-40 from ShinEtsu, constant stress at 100% strain can be measured after 35 days. No definite trend can be observed within 200 days for the post-cured LSR samples.

**Figure 11:**
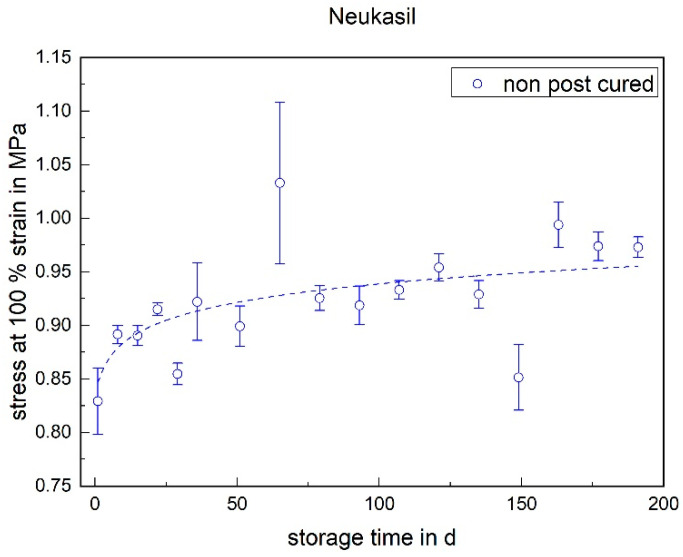
Stress at 100% strain of Neukasil RTV as a function of the storage time.

Like the elongation at break and tensile strength, the RTV shows an increase in stress at 100% strain within the first week (cf. Figure 11). After the first week, a further increase in stress at 100% strain can be observed over the storage period of 200 days. However, this is no longer as pronounced as during the first week. The larger standard deviation in the RTV compared to the LSR is due to the sample preparation. Due to the potting of the plates, the cross-sectional area varies more than with the injection-molded LSR samples.

### 3.2. Shore A Hardness

**Figure 12:**
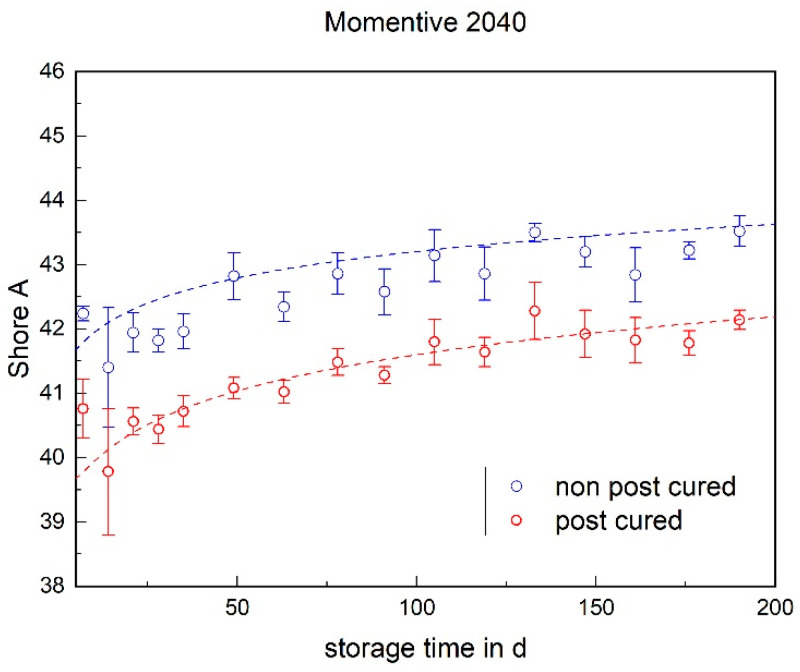
The Shore A hardness of Momentive LSR 2040 as a function of the storage time.

**Figure 13:**
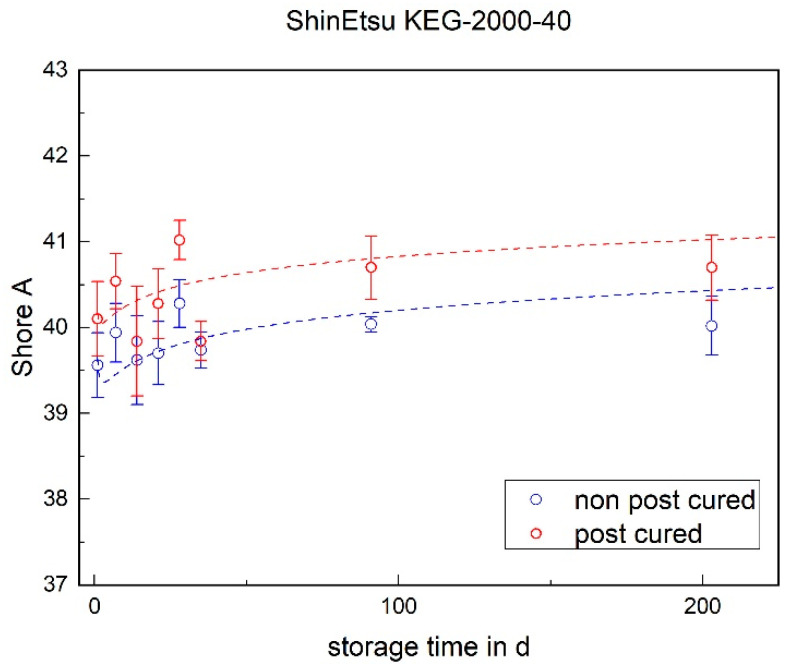
The Shore A hardness of ShinEtsu KEG-2000-40 as a function of the storage time.

The Shore A hardness of both LSRs increases over the storage time (cf. Figure 12 and Figure 13). This increase can be observed in both the post-cured and non-post-cured samples. It is noticeable that the Shore A hardness of the non-post-cured samples of Momentive 2040 is higher than that of the post-cured samples and that the post-cured samples of ShinEtsu KEG-2000-40 have a higher hardness than the non-post-cured samples. To find an explanation for this effect, in-depth chemical analyses have to be carried out. Regardless of the LSR and tempering condition, it is noticeable that the increase in hardness is more pronounced within the first 100 days. A plateau seems to form.

**Figure 14:**
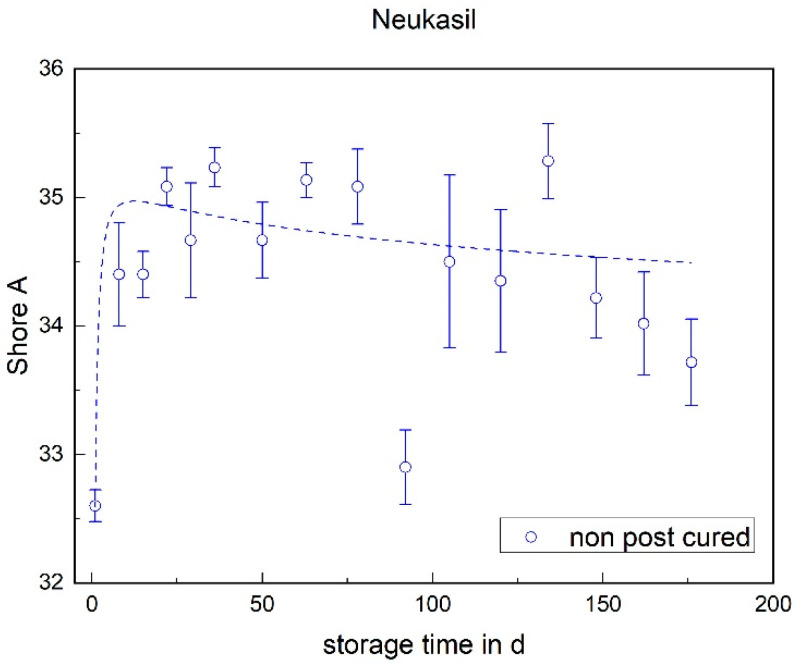
The Shore A hardness of Neukasil RTV as a function of the storage time.

The RTV behaves differently (cf. Figure 14). Here, comparable to the tensile test, a significant increase in the Shore A hardness can be determined within the first week. Over the storage period, however, the hardness slowly decreases again.

### 3.3. Rebound Elasticity

**Figure 15:**
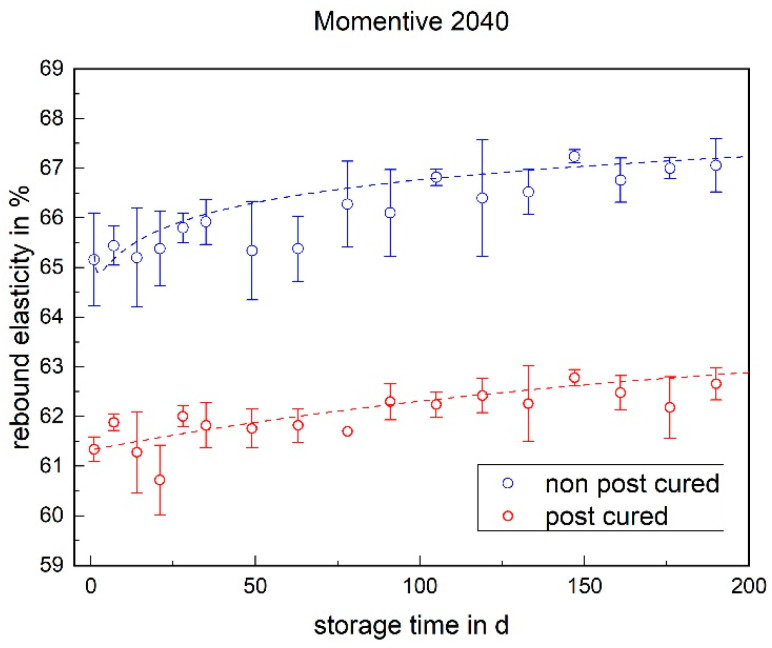
The rebound elasticity of Momentive LSR 2040 as a function of the storage time.

**Figure 16:**
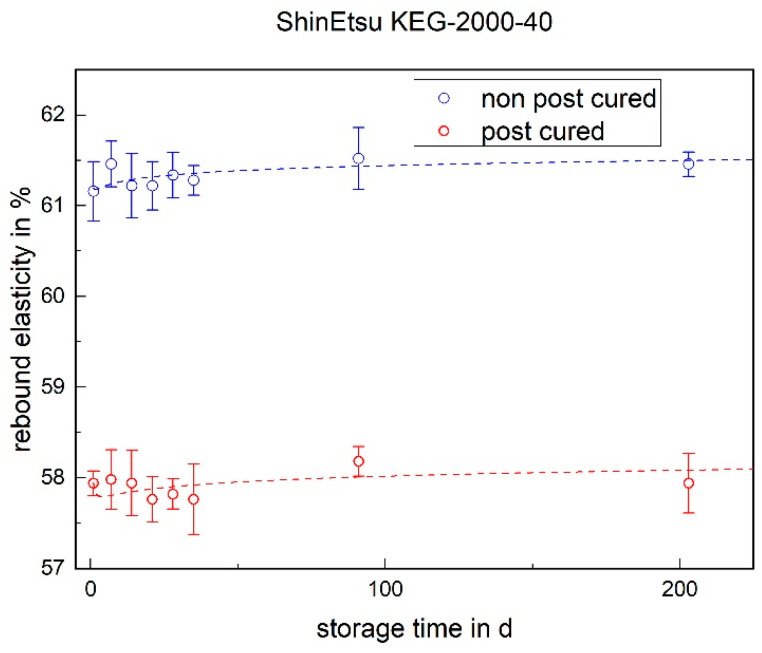
The rebound elasticity of ShinEtsu KEG-2000-40 as a function of the storage time.

Regarding the rebound elasticity, the values of the non-post-cured LSR samples are higher than those of the post-cured samples (cf. Figure 15 and Figure 16). This means that the non-post-cured samples release more energy and therefore have higher elasticity. With the Momentive 2040, the rebound elasticity of both the post-cured and non-post-cured samples increases over the storage time. The ShinEtsu KEG-2000-40 behaves differently, with no significant change in the rebound elasticity over 200 days.

**Figure 17:**
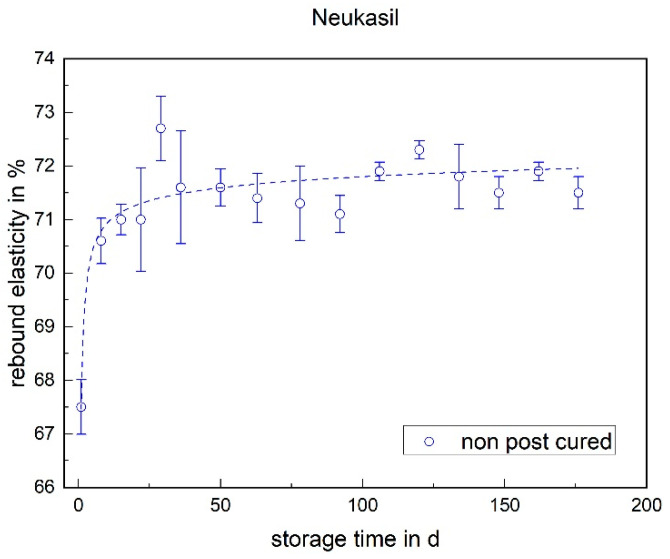
The rebound elasticity of Neukasil RTV as a function of the storage time.

With the Neukasil RTV, a significant increase can again be seen within the first week (cf. Figure 17). After that, a slight trend towards a higher rebound elasticity can be observed over the storage period.

### 3.4. Compression Set

**Figure 18:**
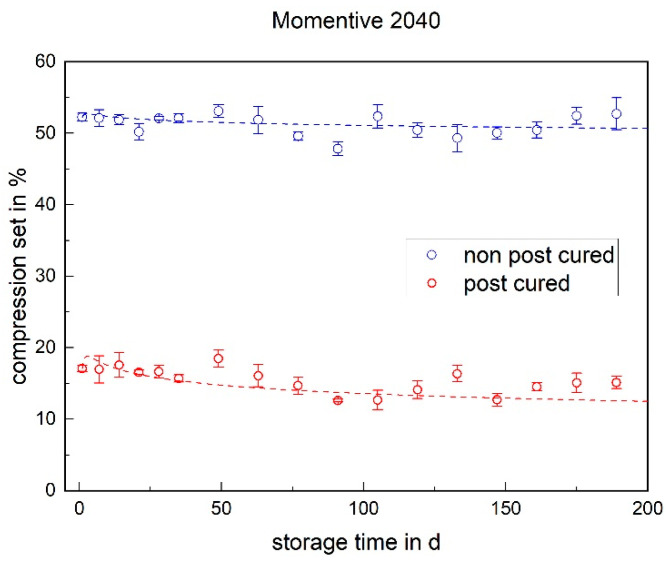
The compression set of Momentive LSR 2040 as a function of the storage time.

**Figure 19:**
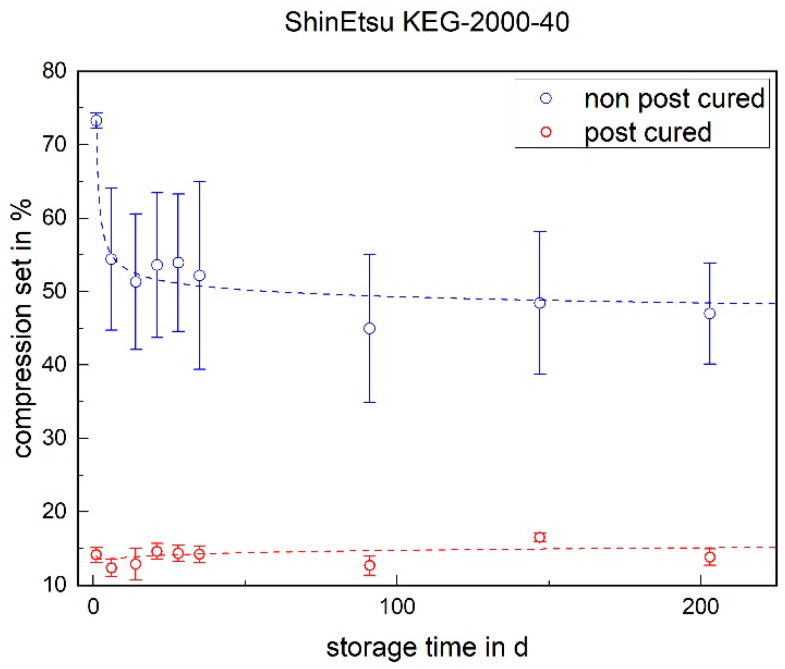
The compression set of ShinEtsu KEG-2000-40 as a function of the storage time.

The compression set indicates the recovery behavior after compression at a defined time and temperature. A low value indicates good recovery behavior. For both LSRs, it is noticeable that the non-post-cured samples have a significantly higher compression set than the post-cured samples (cf. Figure 18 and Figure 19). This can be explained by post-cross-linking. Due to the higher temperature during post-curing, additional cross-linking sites are formed, which are still available in the non-post-cured samples. Due to the compression and storage at 175 °C during the test, these cross-linking sites form in the deformed state of the non-post-cured samples, and recovery after the test is restricted.

With the Momentive 2040, no significant change can be determined over the aging process. This applies to both the post-cured and non-post-cured specimens. The situation is different with the ShinEtsu KEG-2000-40, where a significant decrease can be determined for the non-post-cured samples within the first week. There is no change in the compression set for the post-cured samples.

**Figure 20:**
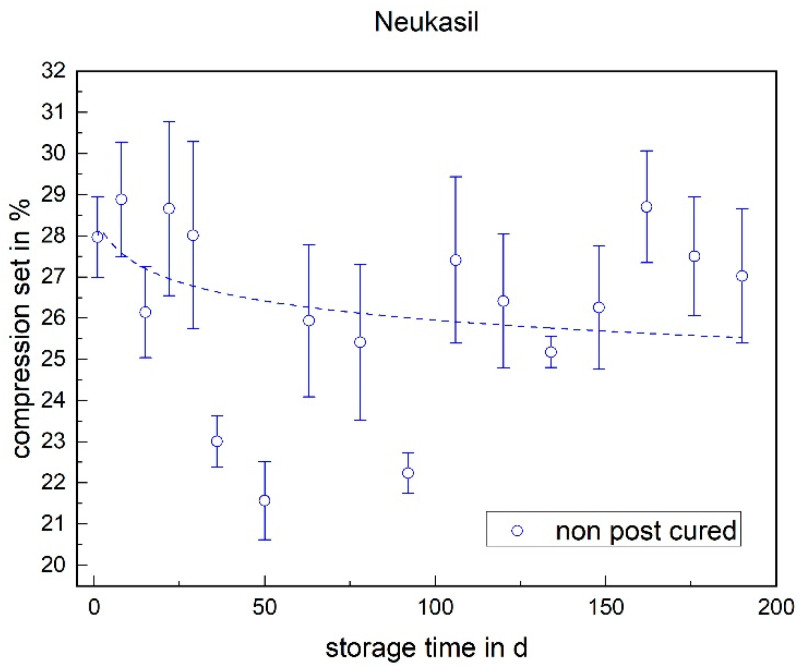
The compression set of Neukasil RTV as a function of the storage time.

No definite trend over the storage time can be determined for the Neukasil RTV, as the variation is very high (cf. Figure 20). Here, it is necessary to increase the storage time or the sample size to be able to conclude the compression set.

### 3.5. H-Tensile Test

**Figure 21:**
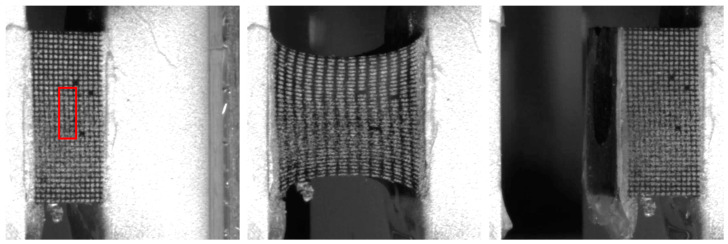
H-Tensile test states obtained using image correlation method (evaluation area in red).

To better reflect the application range (automotive) of the silicone materials, the test and corresponding simulation were carried out only in the strain range of up to 100%. Figure 21 shows the condition of the silicone specimen at 0% and 100% elongation and failure. In the region of the bond, no free strain was possible for the material. Only after a transition zone can the material develop its elongation capability (red marking), and the evaluation then starts in this area of free elongation. Up to an elongation of 100%, neither cohesive nor adhesive failure occurred. In practice, the H sample could stretch up to the range of 150 to 300%. Adhesive failure (tearing off in the region of the bond to the aluminum plate) occurred here earlier than cohesive failure.

**Figure 22:**
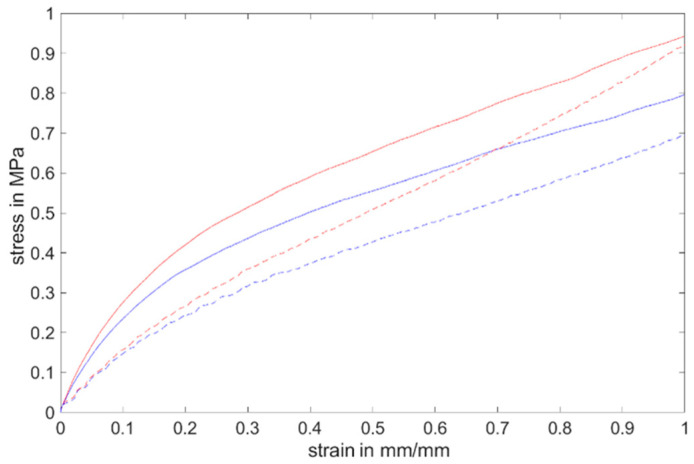
The stress–strain curve on the first test day (red: post-cured Momentive Silopren LSR 2040; blue: non-post-cured Momentive Silopren LSR 2040; continuous: H-test; straight: uniaxial tensile test) (the preload 0.1 MPa has been removed).

Figure 22 shows the stress–strain diagram (mean values) of Momentive Silopren LSR 2040 from the uniaxial tensile test and H-Tensile test on the first test day. The stress–strain curves follow a non-linear progression. The curve from the H sample shows a steeper slope up to a strain of about 30% compared to the uniaxial tensile test, after which the H curves converge with the curves from the uniaxial tensile test. In comparison, the curves from the uniaxial tensile test go rather quickly to the region where tensile acts directly on the strong main bonds of the monomers. Overall, higher stress must be applied for the H sample for the same strain compared to that applied for the uniaxial tensile test. According to [43], the reason for the different behavior is that the H curves follow a distinct unraveling region of the silicone material, where secondary bonds between the long-chain monomers have strong resistance to chain unraveling.

**Figure 23:**
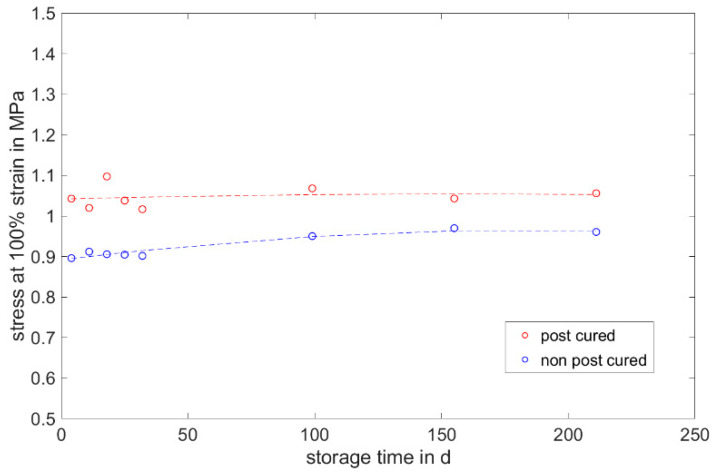
Change in mechanical properties of Momentive Silopren LSR 2040 during real-time storage.

**Figure 24:**
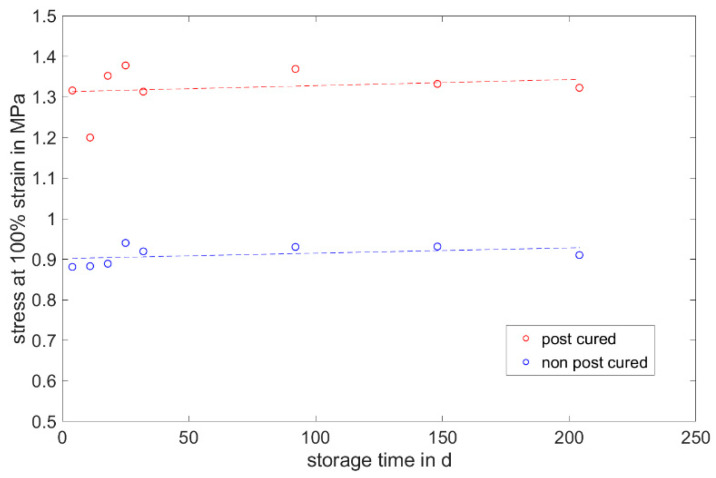
Change in mechanical properties of ShinEtsu KEG-2000-40 during real-time storage (departure at post-cured samples on 11th test day).

**Figure 25:**
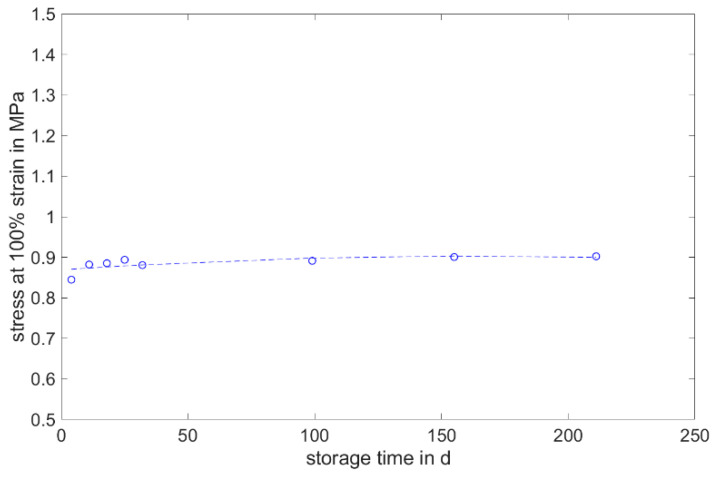
Change in mechanical properties of Neukasil RTV 230 during real-time storage.

The following diagrams (Figure 23, Figure 24 and Figure 25) show the changes in the mechanical properties in the aging process. For all materials, the tests were stored and performed for over 200 days. The course of the stress at 100% strain remains unchanged. Real-time storage has no significant effect on the stress at 100% strain of one form of the H specimen over 200 days. It shows similar changes in the mechanical property as the uniaxial tensile test. For both LSRs, the 100% strain stress of non-post-cured samples increases in the first month, with Momentive’s LSR showing a more pronounced increase in stress that lasts for up to about 148 days. With ShinEtsu’s LSR, constant stress at 100% strain was measured after 32 days. For the post-cured LSR samples, a slight increase can be seen with Momentive, while no clear trend can be observed with ShinEtsu. RTV shows an increase in stress at 100% strain during the first week. After the first week, a slight increase in stress at 100% strain is observed during the 200-day storage period.

### 3.6. Simulation

**Figure 26:**
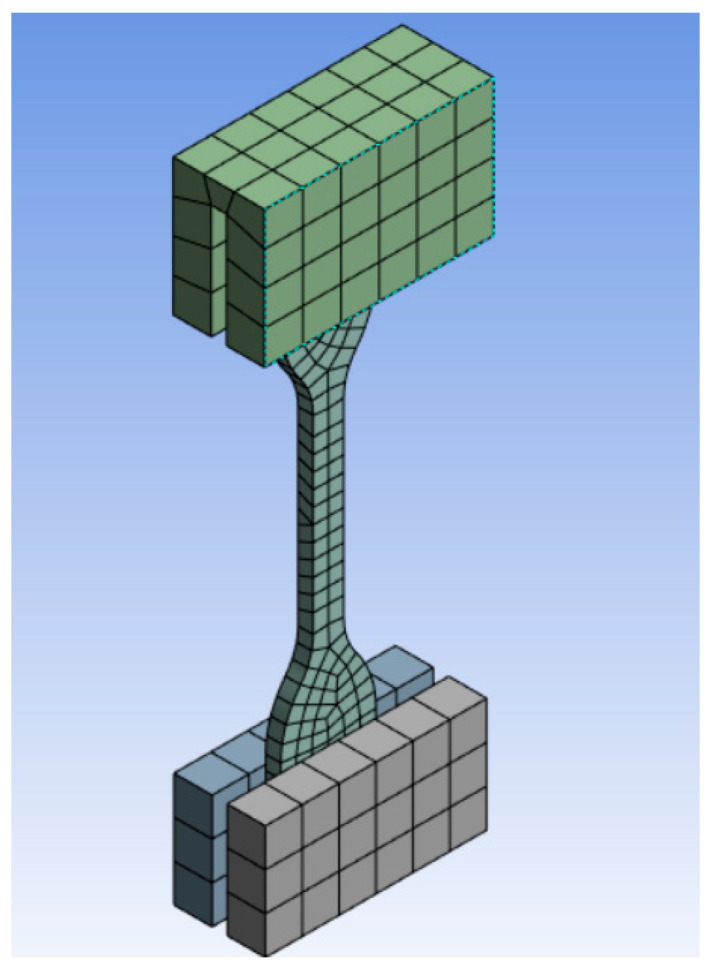
A simulation model of the uniaxial tensile test with mesh.

The numerical calculations of the uniaxial tensile test were performed in the age period of up to 200 days and are compared with the stress–strain curve obtained from the experimental tests. The model setup of the specimen geometry as well as the test rig was modeled in accordance with DIN 53504 [39] (Figure 26). The ends of the specimen clamped by the fixture were simplified here to be bonded to the fixture. The fixture at one end was held stationary, and the fixture at the other end was given a total displacement of 40 mm in the tensile direction. The sample body was meshed with “hex dominant meshing”. Four different element sizes were investigated for meshing, and these four meshes (0.2 mm, 0.5 mm, 1 mm and 2 mm) show very similar accuracy with the root mean square remaining at 0.02. However, the finer the mesh, the more time is required for the computation. The calculations with both 1 mm and 2 mm meshes could be performed in less than a minute (max. utilized cores of Solver: 24). The steel was used for the calculation of the fixture. For the calculation of the silicone elastomers, the Mooney–Rivlin model with three parameters was used. Since the strain ranges up to 100%, the Mooney–Rivlin model can give good results. The form of the strain–energy potential for the Mooney–Rivlin model with a three-parameter model is as follows [44]:

(2) $$W=C_{10}\left(I_{1}-3\right)+C_{01}\left(I_{2}-3\right){+C}_{11}\left(I_{1}-3\right)\left(I_{2}-3\right)+\frac{1}{D_{1}}{\left(J-1\right)}^{2}$$

where W represents the strain energy density; $C_{10},C_{01}\;and\;C_{11}$ are coefficients; $D_{1}$ is the coefficient for compressibility and *I* represents three invariants of the left (or right) Cauchy–Green deformation tensor, which is defined as follows:

(3) $$I_{1}=\lambda_{1}^{2}+\lambda_{2}^{2}+\lambda_{3}^{2}$$

(4) $$I_{2}=\lambda_{1}^{2}\lambda_{2}^{2}+\lambda_{2}^{2}\lambda_{3}^{2}+\lambda_{1}^{2}\lambda_{3}^{2}$$

(5) $$I_{3}=J^{2}=\lambda_{1}^{2}\lambda_{2}^{2}\lambda_{3}^{2}$$

$\lambda_{i}$ represents the stretch ratio in the I direction. Ni [45] has shown that compressibility can be ignored for the materials used in this project. For the uniaxial deformation mode, the incompressibility constraint yields the following:

(6) $$\lambda_{2}=\lambda_{3}=\frac{1}{\sqrt{\lambda_{1}}}$$

The engineering stress can be derived from a derivative of the strain energy density function by the stretch ratio [46].

(7) $$\sigma_{i}=\frac{\partial W}{\partial \lambda_{i}}=\frac{\partial W}{\partial I_{1}}\frac{\partial I_{1}}{\partial \lambda_{i}}+\frac{\partial W}{\partial I_{2}}\frac{\partial I_{2}}{\partial \lambda_{i}}$$

The engineering stresses derived from the Mooney–Rivlin model depend on both the first invariant and second invariant. By substituting the strain energy density model in Equation (7), the engineering stress can be expressed as follows [46]:

(8) $$\sigma =2C_{10}\left(\lambda -\lambda^{-2}\right)+2C_{01}\left(1-\lambda^{-3}\right)+6C_{11}(\lambda^{2}-\lambda -1+\lambda^{-2}+\lambda^{-3}-\lambda^{-4})$$

**Figure 27:**
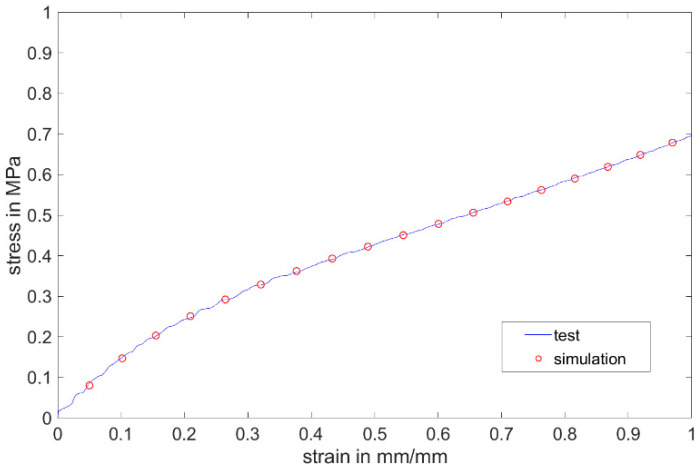
A comparison between the test and simulation on the first test day with non-post-cured Momentive Silopren LSR 2040.

The data obtained by the uniaxial tensile test were inserted into the Mooney–Rivlin model. The stress–strain curve from the simulation show very good agreement with the test curve in the age period of up to 200 days (Figure 27).

**Figure 28:**
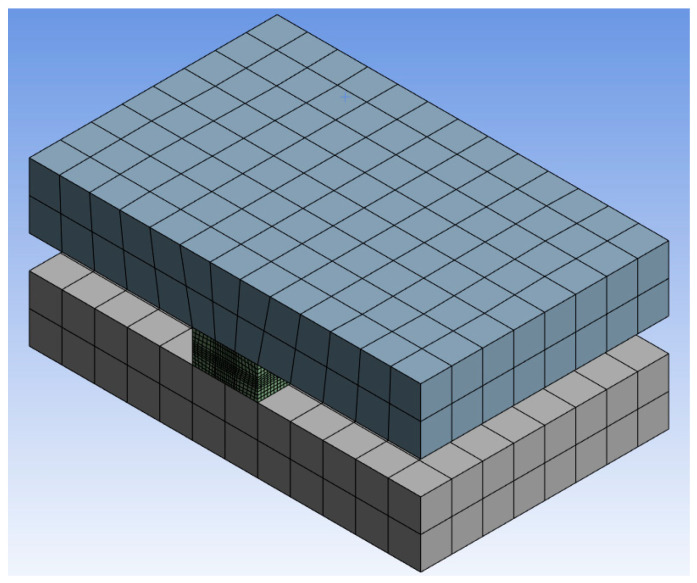
A simulation model of the H-test with mesh.

**Figure 29:**
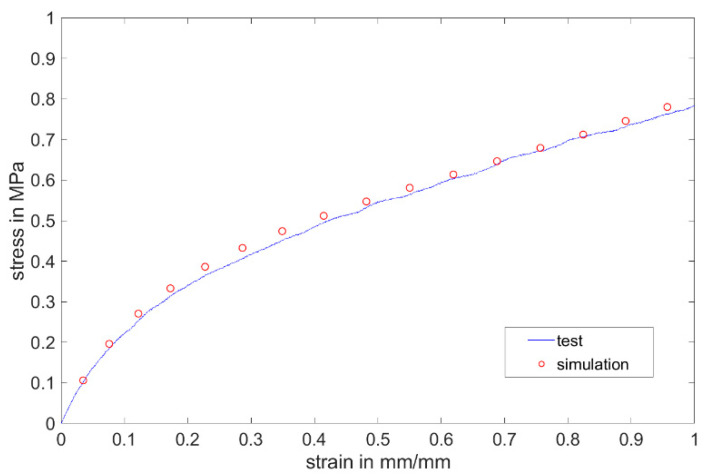
Comparison between test and simulation of stress–strain curve on first test day with non-post-cured Momentive Silopren LSR 2040.

**Figure 30:**
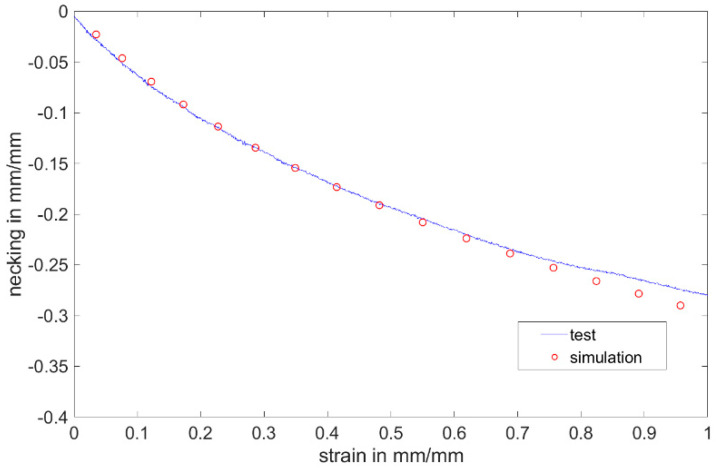
Comparison between test and simulation of necking–strain curve on first test day with non-post-cured Momentive Silopren LSR 2040.

Numerical calculations of the H-test for non-post-cured LSR up to 200 days were carried out. The model setup of the specimen geometry as well as the test rig was modeled after the setup of the experimental tests with all boundary conditions that need to be considered (Figure 28). The specimen was bonded to the fixture. The fixture at one end was held stationary, and the fixture at the other end was given a total displacement of 4 mm in the tensile direction. The sample body was meshed with two different mesh methods, uniform meshing of the entire specimen or finer meshing in the evaluation polygon. The computational accuracy of the two methods was very similar, with the latter reducing the computational time by more than half compared to the former. Four different element sizes (0.05 mm, 0.1 mm, 0.2 mm and 0.5 mm) were investigated for meshing, and the first three meshes show very similar accuracy with the root mean square remaining at 0.03. The following meshing for the specimen was finally chosen in the evaluation with the element size of 0.2 mm and other regions with the element size of 0.5 mm. Aluminum was used for the calculation of the fixture. The data verified by the simulation of the uniaxial tensile test were inserted into the Mooney–Rivlin model. The stress–strain curve and necking–strain curve from the simulation show very good agreement with the test curve in the age period of up to 200 days (Figure 29 and Figure 30). It shows that for the non-post-cured LSR material, the coefficient obtained from the uniaxial tensile test can be transferred to other geometry.

**Figure 31:**
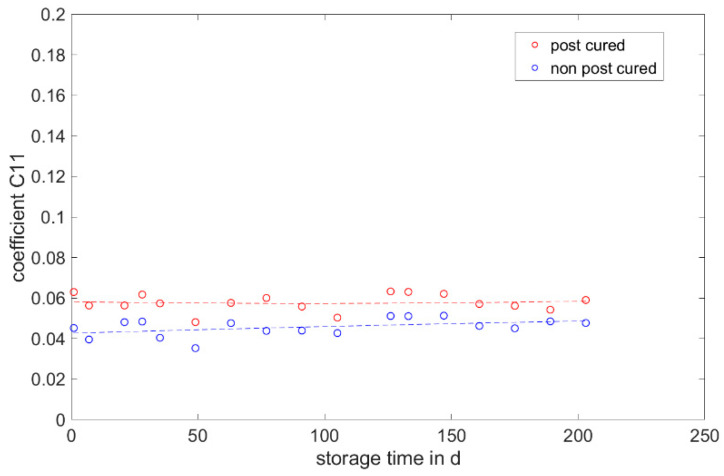
Change in material coefficient $C_{11}$ of Momentive Silopren LSR 2040 during real-time storage.

**Figure 32:**
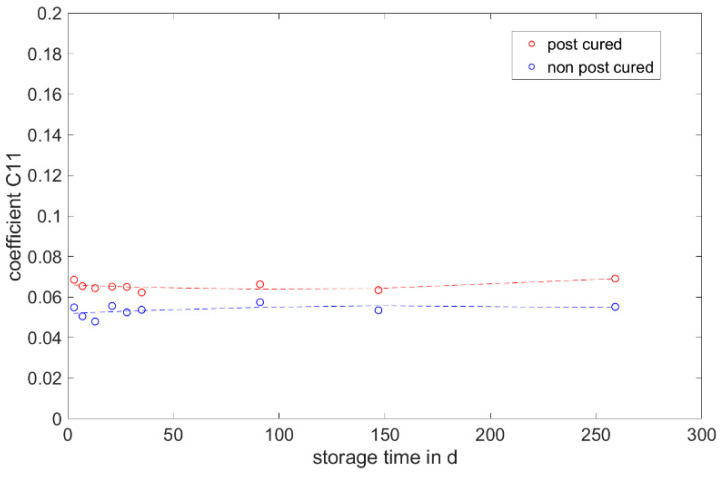
Change in material coefficient $C_{11}$ of ShinEtsu KEG-2000-40 during real-time storage.

**Figure 33:**
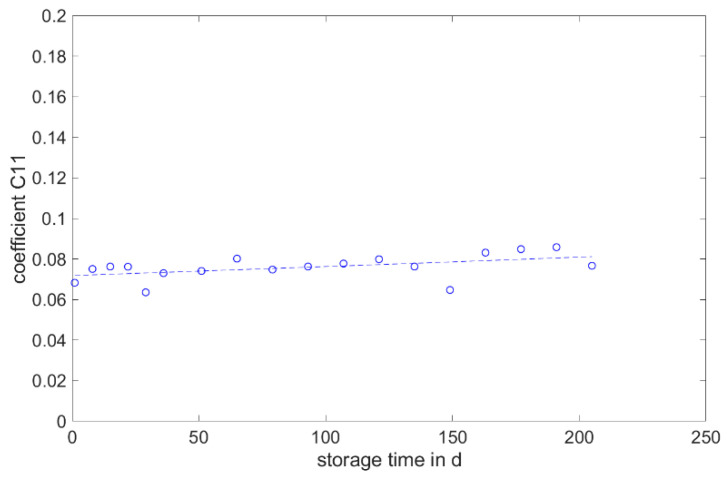
Change in material coefficient $C_{11}$ of Neukasil RTV 230 during real-time storage.

The determined and validated material coefficients from the uniaxial tensile test were used for the aging process. The focus of the investigation here is on $C_{11}$, from Equation (2), which has the highest influence on the stress–strain curve. The material coefficient $C_{11}$ is shown in Figure 31, Figure 32 and Figure 33. Due to the high agreement between the $C_{11}$ trend over time and the change in stress at 100% strain in the tensile test (Figure 9, Figure 10 and Figure 11) and H-test (Figure 23, Figure 24 and Figure 25), it can be seen that $C_{11}$ can represent the tensile and elastic properties of the material under study, and the changes in $C_{11}$ over time can show the aging effect of the elastic properties of the material under study. A comparison of the post-cured and non-post-cured LSR materials shows that the $C_{11}$ coefficient of the post-cured material is higher than the non-post-cured material throughout the aging process. The larger $C_{11}$ coefficient is indicative of an increased degree of cross-linking of the material. It can be seen through the aging process of all the materials that an increase in the $C_{11}$ coefficient may indicate an increase in the degree of cross-linking of the material or higher internal stresses during the aging process. The aging process leads to an increase in cross-linking reactions. These cross-linking reactions may make the material harder but also more brittle. The aging process of hardness (Figure 12, Figure 13 and Figure 14) also confirms this argument and the correlation with $C_{11}$. As the material ages, the internal structure may change, leading to the accumulation of internal stresses. These internal stresses may lead to earlier damage to the material under a load. Changes in the $C_{11}$ coefficient not only reflect the current state of a material’s mechanical properties, but also provide an important basis for predicting future changes in material properties. This enables the use and maintenance of materials so that they can be managed more effectively through the real-time monitoring and analysis of $C_{11}$ coefficients, ensuring structural safety and reliability.

## 4. Discussion

The aging tests revealed that the mechanical properties of silicones may alter over time. Compared to previous publications, the changes in the mechanical parameters were determined over a significantly longer period. In addition, the results for additive curing silicone elastomers are presented here, as many publications deal with condensation curing or peroxide curing systems. However, the extent of the change depends on the type of silicone (LSR or RTV) and the aging conditions. Thus, it was shown that despite the same curing mechanism being used, the curing temperature influences the change in properties over the storage time.

In general, both LSRs, Momentive Silopren LSR 2040 and ShinEtsu KEG-2000-40, exhibited increases in the Shore A hardness and rebound elasticity, while the tensile strength remained relatively constant throughout the aging period. The post-curing process had a significant impact on the stress at 100% strain and with the compression set. Both values remained stable over time, but the non-post-cured samples exhibited a lower compression set and higher stress at 100% strain. These differences are due to post-curing during tempering.

The increase in the Shore A hardness is an indication of post-cross-linking during the storage period. This can also be easily seen in the stress at 100% strain in the non-post-cured samples.

The Altropol RTV exhibits a different trend compared to the LSRs, with the tensile strength and Shore A hardness increasing in the first week before slightly decreasing, while the compression set decreases in the first weeks before slightly increasing. The stress at 100% strain increases over the aging period, while the compression set decreases.

The increase or decrease in the characteristic values within the first week is due to post-cross-linking. Although the silicone elastomer is demoldable after 24 h, it is not yet fully cross-linked. This does not appear to be achieved with this RTV until after approx. one week. The increase in the Shore A hardness of approx. 2 Shore A corresponds well with the increase within the first 7 days mentioned in the datasheet [36]. The decreases in the tensile strength and elongation at break with a longer storage time can also be explained by post-cross-linking. It is generally known that the tensile strength of elastomers first increases with an increasing cross-linking density and then decreases again after reaching maximum strength. The elongation at break decreases as the degree of cross-linking increases. However, the decrease in the hardness of the RTV over the storage period cannot be explained by post-cross-linking. It is not possible to determine the degree of cross-linking with the accuracy required here using the current state of the art. Swelling tests and similar test methods have measurement inaccuracies that are too high. Therefore, the focus of this publication is not on determining the aging mechanism but on the change in properties over time.

To reveal aging phenomena in the coefficients of the Mooney–Rivlin model, an FEM model was parameterized with the tensile test in accordance with DIN 53504, in addition to the standardized test methods.

It is noticeable that coefficient $C_{11}$ shows the greatest change in the coefficients over the storage time. The change shows a good correlation with the development of stress at 100% strain over the aging period.

This study aims to establish a correlation between the change in mechanical properties and the parameters of the Mooney–Rivlin model. No correlation is found between the Shore A hardness, tensile strength and elongation at break, DVR and rebound resilience and the parameters. However, the parameter $C_{11}$ shows a good correlation with the value of stress at 100% strain. Changes in the $C_{11}$ coefficient not only reflect the current state of a material’s mechanical properties but also provide an important basis for predicting future changes in material properties.

In future work, the Mooney–Rivlin model can be extended with additional time-dependent terms to describe the Mooney–Rivlin parameters during aging. Also, the accuracy of long-term predictions can be improved by increasing the time of aging. The effect of different storage media on the aging and mechanical properties of LSRs and RTVs can also be investigated to help determine the optimal storage conditions.

## Figures and Tables

**Table 1:** The manufacturing parameters in the injection molding process [39,40].

Material	Geometry	Standard	Mold Temperature in °C	Vulcanization Time in s	Injection Volume in cm^3^
Momentive Silopren LSR 2040	Tensile bar S2	DIN 53504	180	8	3
	cylindrical disk type A	DIN ISO 815-1	180	105	10.5
	cylindrical disk type B	DIN ISO 815-1	180	25	2.25
	H sample	-	130	58	3
ShinEtsu KEG-2000-40	Tensile bar S2	DIN 53504	180	8	3
	cylindrical disk type A	DIN ISO 815-1	180	110	9.6
	cylindrical disk type B	DIN ISO 815-1	180	35	2.15
	H sample	-	130	58	3.35

**Table 2:** The parameters of the mixing program of the RTV.

Program Level	Revolutions in rpm	Time in min	Pressure in mbar
1	1000	00:15	1000
2	800	01:00	1000
3	800	01:00	500

## Data Availability

The original contributions presented in the study are included in the article, further inquiries can be directed to the corresponding authors.
